# BAG3 in traumatic brain injury: A cell-type-specific modulator of tau hyperphosphorylation

**DOI:** 10.4103/NRR.NRR-D-25-00503

**Published:** 2025-09-03

**Authors:** Nicholas Sweeney, Tae Yeon Kim, Hongjun Fu

**Affiliations:** Department of Neuroscience, College of Medicine, The Ohio State University, Columbus, OH, USA; Biomedical Sciences Graduate Program, College of Medicine, The Ohio State University, Columbus, OH, USA; Chronic Brain Injury Institute, The Ohio State University, Columbus, OH, USA

**BCL2-associated anthanogene 3 facilitates the clearance of tau protein aggregates:** BCL2-associated anthanogene 3 (BAG3) is a ubiquitously expressed and highly conserved multi-functional co-chaperone protein involved in many biological processes that supports cellular homeostasis, including the inhibition of apoptosis by preventing mitochondrial BAX localization (Lin et al., 2022) and the promotion of the degradation of hyperphosphorylated tau aggregates by its interactions with SQSTM1 (p62) (Hamano and Mutoh, 2022).

BAG3 and its interacting proteins harness the capability to direct the clearance of hyperphosphorylated tau aggregates via the autophagy-lysosome pathway (ALP) and the endosomal lysosome pathway (Lin et al., 2022). A previous study indicates that BAG3 interactions with synaptopodin in the post-synaptic densities of neurons facilitate tau clearance through modulation of the ALP (Lin et al., 2022). Disruption of either synaptopodin or BAG3 resulted in aberrant tau accumulation in autophagosomes in post-synaptic terminals (Lin et al., 2022). Overexpression of BAG3 in mouse primary cortical neurons reduced hyperphosphorylated tau accumulation, while its knock-down enhanced tau hyperphosphorylation (Fu et al., 2019). Furthermore, BAG3 interacts with TBC1 Domain Family Member 10B (TBC1D10B) to facilitate tau clearance through the endosomal lysosome pathway (Lin et al., 2022). The trimer composed of BAG3-Hsp70-TBC1D10B prevents the inactivation of Rab35 by TBC1D10B, leading to the initiation of endosomal sorting complexes required for transport-mediated tau clearance (Lin et al., 2022). Further support of this mechanism was found *in vivo* in the P301S model of tauopathy (PS19), where overexpression of BAG3 enhanced its colocalization with the endosomal sorting complexes required for transport-related protein CHMP2B, leading to the reduction of accumulated tau species (Lin et al., 2022). Thus, in the context of Alzheimer’s disease (AD) and other tauopathies, BAG3 is a co-chaperone capable of maintaining the cellular homeostasis via the clearance of tau species through either the ALP or endosomal lysosome pathway.

Aside from AD, BAG3 has been implicated in many other diseases, such as cancer (Lin et al., 2022), dilated cardiomyopathy (Qu and Hakonarson, 2024), and Parkinson’s disease (Sheehan et al., 2023), highlighting the importance of the role of BAG3 in maintaining cellular homeostasis in a wide range of diseases. However, the role of BAG3 in clearing hyperphosphorylated tau after traumatic brain injury (TBI) remains elusive.

**The link between TBI and AD: the importance of tau pathology:** Epidemiological studies suggest that a single TBI can increase the risk of developing AD in patients over 55 years old compared to those who experience non-TBI trauma, with an impact of both TBI severity and age categorization (Gardner et al., 2014). Despite the mounting epidemiological evidence linking TBI and AD, other studies challenge this association, such that experiencing TBI with loss of consciousness was not predictive of the presence of neuritic plaques or neurofibrillary tangles (Crane et al., 2016), the two main pathological hallmarks of AD. The discrepancies between TBI and AD risk in the literature highlight the need for an improved understanding of the molecular mechanisms underlying the relationship between TBI and AD.

Insights into the etiology of the link between TBI and AD can be ascertained from chronic traumatic encephalopathy (CTE), a disease characterized by repetitive head impacts. CTE is widely accepted as a distinct tauopathy, with a unique patterning of neuropathologic lesions consisting of hyperphosphorylated tau at sulci depths and superficial cortical layers, amyloid beta accumulation, and cytosolic TDP-43 mislocalization (McKee et al., 2023). The classification of CTE as a unique tauopathy emphasizes the importance of focusing on tau pathology as the primary causative neuropathologic hallmark resulting in cognitive dysfunction after a single or repetitive head injury. Still, a significant gap exists in the literature focusing on tau pathology as a bridge between TBI and AD-related neuropathologic change.

Few studies have examined how cell-type-specific disruptions of protein clearance mechanisms may contribute to tau hyperphosphorylation and aggregation. ALP dysfunctions increase throughout aging and are exacerbated in AD (Hamano and Mutoh, 2022). Thus, tau pathology and disrupted ALP functions may provide the missing link between TBI and AD.

While BAG3 has been previously reported to play a role in tau hyperphosphorylation and aggregation (Fu et al., 2019), the role of BAG3 in the context of TBI remained largely unexplored. Using pre-clinical mouse models of TBI and post-mortem human brain tissue, we show that BAG3 deficiency and downstream ALP dysfunction are eminently linked to the accumulation of hyperphosphorylated tau after TBI. Overexpression of human BAG3 into hippocampal neurons before TBI ameliorated tau hyperphosphorylation, synaptic dysfunction, and memory deficits likely through modulation of the ALP (Sweeney et al., 2024).

**BAG3 level as a mediator of cell-type-specific tau inclusions after TBI:** Previous reports indicate the differential expression of BAG3 in excitatory and inhibitory neurons and astrocytes in AD (Fu et al., 2019), suggesting that BAG3 may contribute to intrinsic vulnerability to aberrant tau accumulation. Building upon this evidence, we were curious whether differential BAG3 protein levels may contribute to tau accumulation in TBI.

Notably, the pre-clinical TBI data are mixed, with some studies suggesting increased tau hyperphosphorylation after TBI, and others citing no significant change. Thus, proper characterization of our controlled cortical impact (CCI) TBI animal models and the post-mortem human brain tissue was imperative to draw meaningful conclusions about the effect of TBI on AD-associated neuropathologic changes. In wild-type (C57BL/6J) and human tau knock-in mice (Saito et al., 2019), we found that a single CCI TBI resulted in increased tau hyperphosphorylation in excitatory neurons and oligodendrocytes (Sweeney et al., 2024). This increased tau hyperphosphorylation coincided with increased astro- and micro-gliosis in regions proximal and distal to the injury site, post-synaptic dysfunction, and memory deficits as assessed by the Y-Maze (Sweeney et al., 2024). Immunofluorescence staining and Western blot assay of the inferior parietal lobule of post-mortem human brain tissue revealed increased tau hyperphosphorylation and gliosis in cases with a history of TBI and AD compared to neuropathologically negative controls (Sweeney et al., 2024), suggesting that our pre-clinical models were sufficient to ascertain the mechanisms underlying tau hyperphosphorylation after TBI.

We next sought to determine how the cell-type-specific protein level of BAG3 is perturbed following TBI. In the mouse models and post-mortem human brain tissue, we found stark upregulation of astrocytic BAG3, while neurons and oligodendrocytes burdened with hyperphosphorylated tau exhibited decreased levels (Sweeney et al., 2024). Thus, dysfunctional BAG3 homeostasis may underlie the cell-type-specific vulnerability to tau hyperphosphorylation after TBI.

Overexpression of human BAG3 into hippocampal neurons before TBI ameliorated tau hyperphosphorylation, post-synaptic density loss, and cognitive deficits on hippocampal-dependent memory tasks such as the Y-Maze and Morris Water Maze (Sweeney et al., 2024). Based on the pre-established role of BAG3 as a modulator of autophagic flux (Hamano and Mutoh, 2022; Lin et al., 2022), we hypothesized that BAG3 deficiency after TBI caused ALP deficits, while its overexpression would increase ALP function, leading to improved pathological and behavioral outcomes. In an *in vitro* system, we modulated the level of BAG3 in HEK293 cells co-transfected with an LC3 reporter virus (FUW-mCherry-GFP-LC3), showing that modulation of BAG3 directly impacts ALP dynamics (Sweeney et al., 2024). Turning back to the CCI TBI mouse models and post-mortem human brain tissue, we found global ALP dysfunction as marked by decreased lysosomal markers (cathepsin D and lysosomal associated membrane protein 1) and increased p62 aggregates (Sweeney et al., 2024). Importantly, overexpression of human BAG3 in hippocampal neurons before TBI ameliorated the ALP deficits observed after CCI TBI, indicating that enhancement of BAG3 levels in neurons was sufficient to increase ALP function, likely leading to the observed decreased tau hyperphosphorylation and behavioral deficits after TBI (Sweeney et al., 2024).

**Figure 1:**
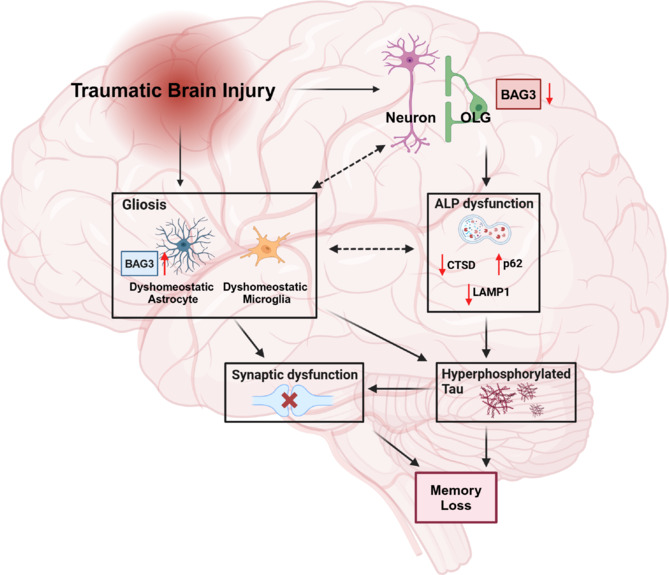
Role of BAG3 in the cell-type-specific vulnerability to tau hyperphosphorylation after traumatic brain injury. Schematic diagram indicating the role of BAG3 in tau hyperphosphorylation, autophagy-lysosome pathway dysfunction, gliosis, and synaptic dysfunction after TBI. Created with BioRender.com. ALP: Autophagy-lysosome pathway; BAG3: BCL2-associated anthanogene 3; CTSD: cathepsin D; LAMP1: lysosomal associated membrane protein 1; OLG: oligodendrocyte; p62: SQSTM1.

Thus, our data suggest that TBI results in the downregulation of BAG3 in neurons and oligodendrocytes because of cell-autonomous or non-cell-autonomous (i.e., gliosis) mediated mechanisms. Decreased BAG3 in neurons and oligodendrocytes results in dysfunction of the ALP (reduced cathepsin D and lysosomal associated membrane protein 1 and increased p62 aggregates), which promotes the accumulation of hyperphosphorylated tau. The hyperphosphorylated tau may also be exacerbated by the robust pro-inflammatory environment of TBI, mediated by the activation of microglia and astrocytes. The combination of gliosis and tau accumulation may contribute to long-term post-synaptic dysfunction, which in tandem with tau-mediated neuronal cell death (likely via necroptosis) causes cognitive dysfunction and memory deficits (**Figure 1**). Therapeutic intervention via BAG3 modulation may inhibit this complex molecular cascade, reducing tau accumulation and improving memory deficits following TBI.

**Targeting BAG3 in TBI and neurodegeneration: emphasis on cell-type-specific modulation:** Our findings highlight the importance of cell-type-specific regulation of BAG3 in TBI and AD. Cell types with reduced BAG3 levels may have an intrinsic vulnerability to hyperphosphorylated tau inclusions (i.e., neurons and oligodendrocytes), and cell types with increased BAG3 levels (i.e., astrocytes) may be protected from proteinaceous inclusions (Sheehan et al., 2023; Sweeney et al., 2024).

Previous investigations to determine the role of BAG3 in the clearance of protein aggregates also establish the importance of the cell-type-specific regulation of BAG3. Sheehan et al. (2023) showed that conditional knock-out of BMAL1 (a circadian clock gene) significantly increased astrocytic BAG3. The upregulation of BAG3 significantly reduced MC1^+^ tau pathology in P301S tau transgenic mice, alpha-synuclein seeding, and microglial reactivity (Sheehan et al., 2023). Specific overexpression of astrocytic BAG3 also reduced tau and alpha-synuclein seeding, likely via astrocyte-mediated uptake (Sheehan et al., 2023). Interestingly, recent genome-wide association studies have identified BAG3 as a candidate gene in Parkinson’s disease, and a SNP in BAG3 (rs72840788) was associated with an increased risk of Parkinson’s disease development (Sheehan et al., 2023). The rs7240788 SNP corresponds with chromatin accessibility in astrocytes (Sheehan et al., 2023), suggesting that astrocytic dysregulation of BAG3 in Parkinson’s disease may be driving disease risk.

The work by Sheehan et al. (2023) and our own (Sweeney et al., 2024) suggest the cell-type-specific expression of BAG3 must be considered when evaluating BAG3 (or other ALP enhancers) to help facilitate the clearance of protein aggregates in TBI, AD, and other neurodegenerative disorders. The mechanisms surrounding the complexities of BAG3 highlight the need for precision therapeutics in the future to develop novel therapeutics for devastating neurological diseases.

**Conclusions and future perspectives:** While we demonstrate the importance of BAG3 in facilitating the clearance of hyperphosphorylated tau, a few questions remain elusive. Notably, TBI is marked by robust and prolonged activation of microglia and astrocytes, which may contribute to a pro-inflammatory environment detrimental to the chronic TBI condition. While neuron-specific overexpression of BAG3 was insufficient to reduce astro and microgliosis after TBI (Sweeney et al., 2024), it is unclear whether targeting BAG3 to other cell types may confer protection against the gliosis phenotype. Sheehan et al. (2023) suggest targeting astrocytic BAG3 may reduce microglial activation. Exploring this phenomenon in the context of TBI is imperative to understand the cell-type-specific role of BAG3 in TBI.

Further, CTE is characterized by astrocytic tau accumulation, localized to the sulci depths (McKee et al., 2023). Interestingly, our mouse models and human brain tissue show stark upregulation of astrocytic BAG3 after TBI and a lack of astrocytic tau inclusions. Thus, it is imperative to know whether the astrocytes with tau inclusions in patients diagnosed with post-mortem CTE have a deficiency in BAG3, which may contribute to their vulnerability to pathological tau inclusions.

Finally, it is unclear to us how the levels of BAG3 are regulated after TBI. Whether the reduction of BAG3 in neurons and oligodendrocytes acts in a cell-autonomous (i.e., independently) or a cell-non-autonomous manner (i.e., based on communication with other cell types and the environment) remains elusive. A detailed mechanistic understanding of how BAG3 is regulated at a cell-type-specific level will be imperative to develop therapeutic strategies for BAG3 in a cell-type-specific manner, potentially allowing for the identification of upstream regulators of BAG3.

In conclusion, as a regulator of the ALP, BAG3 is an attractive therapeutic target for enhancing protein degradation systems in TBI, AD, and other neurodegenerative diseases. Future delineation of methods to fine-tune the protein level of BAG3 in a cell-type-specific manner presents a novel opportunity to develop improved therapeutics for TBI and other devasting neurodegenerative disorders.

*This work was supported by the award W81XWH1910309 (to HF) from the Department of Defense, the award R01-AG075092-01 (to HF) and the award RF1AG063521 from the National Institute of Aging at the National Institutes of Health, the Neurological Research Institute Seed grant (to HF) from The Ohio State University, and the Summer Undergraduate Research Fellowship (to NS) from The Ohio State University Chronic Brain Injury Discovery Theme.*
